# Virtual platform to tackle challenges associated with lifelong medical education during the COVID-19 pandemic

**DOI:** 10.1186/s12909-024-05686-7

**Published:** 2024-06-26

**Authors:** Sang-Hun Ko, Ki-Bong Park, Jae-Ryong Cha, Young-Dae Jeon, Sang-Gon Kim

**Affiliations:** Department of Orthopedic Surgery, Ulsan University Hospital, University of Ulsan College of Medicine, 25 Daehakbyeongwon-ro, Dong-Gu, Ulsan, 44033 Republic of Korea

**Keywords:** Lifelong medical education, Orthopedics, COVID-19, Virtual platform

## Abstract

**Background:**

During the COVID-19 pandemic, large in-person conferences were mostly cancelled to avoid further disease contagion. Physicians continued to demand changes in form to enable participation in lifelong medical education programs, and the traditional model of in-person conferences needed to be rethought. As such, a regional branch of the national orthopedic association tried to move in-person conferences onto a virtual platform. This study aimed to investigate the effect of transitioning large in-person conferences to a virtual model during the COVID-19 pandemic, especially examining any differences in the attendance of each type of conference.

**Methods:**

In this retrospective observational study, 776 participants in virtual conferences and 575 participants in in-person conferences were analyzed. Institutions were classified based on their location in a central city and two neighboring cities. Affiliated institutions were divided into resident training hospitals, general hospitals, and private clinics. The change in the number and proportion of participants between the virtual conference year and in-person conference year was calculated.

**Results:**

The number of virtual conference participants was significantly greater than that of in-person conference participants (*P* = 0.01). Although the highest number of participants was from central city for both years, the proportion of participants from the two neighboring cities increased. Although the proportion of participants from resident training hospitals and private clinics decreased, the proportion of participants from general hospitals increased.

**Conclusions:**

We implemented a virtual platform to tackle challenges associated with lifelong medical education during the COVID-19 pandemic. The virtual platforms can be helpful for organizations that must hold regular lifelong medical education programs for members spread across a wide geographic region.

## Background

The lockdowns and physical distancing rules implemented in response to the COVID-19 pandemic significantly impacted every aspect of affected people’s social lives [1]. The medical field was similarly affected, with reductions in outpatient clinic visits, postponed elective surgeries, and drastic reductions in the number of surgeries [2, 3]. Regarding education, medical school education, academic meetings, and in-person conferences (IPCs) were greatly reduced or outright banned [4].

After graduating from medical school, all physicians need to continuously maintain, update, or develop their knowledge, skills, and attitudes for their professional practice [5]. Continuing professional development (CPD) is ‘any type of learning that professionals undertake to increase their knowledge, understanding and experiences of a subject area or role’ [6]. Most physicians pursue CPD to better serve their patients and operate a safe and profitable practice [7]. CPD also refers to the continuing development of non- medical competencies including professional, interpersonal, managerial, and communication skills [5]. CPD is typically offered via traditional educational meetings, such as conferences, lectures, workshops, seminars, and symposia. They usually also provide printed educational materials or other resources [6].

Unlike undergraduate, graduate, and postgraduate education, CPD education has no commanding authority or fixed space. Therefore, CPD education is bound to be limited without investment of time and money [8, 9]. With the high demand for CPD and restrictions on IPC during the COVID-19 pandemic, it is necessary to reconsider the traditional model of IPC in the context of virtual conferences (VCs) [10, 11]. However, information on designing and operating a virtual platform for CPD conferences during the COVID-19 pandemic is limited.

This study aimed to investigate the effect of transitioning large IPCs to a virtual model during COVID-19 pandemic, especially examining any differences in the attendance of each type of conference.

## Methods

### Study design and participants

**Fig. 1:**
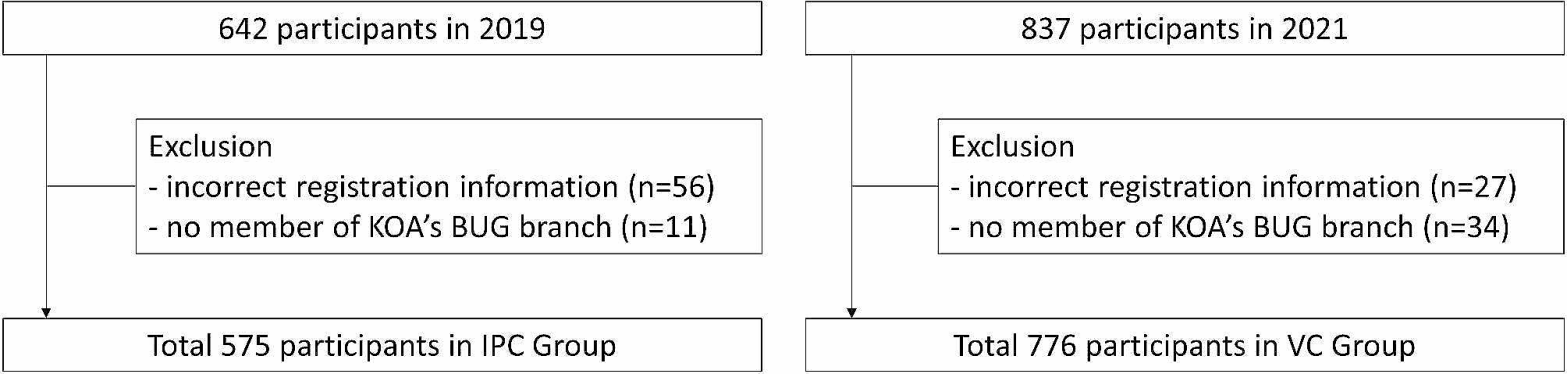
Flowchart of participants in this study

This study was a retrospective observational study of participants of a monthly orthopedic CPD conference held by the Busan-Ulsan-Gyeongnam (BUG) branch between 2019 and 2021 (14 months). It is one of the seven branches of the Korean Orthopaedic Association (KOA) and includes 13 orthopedic resident training hospitals (10 university hospitals and three general hospitals) and other general hospitals and private clinics as member institutions (Table 1) [12]. The exclusion criteria of this study are as follows: participants with missing or incorrect registration information and orthopedic professionals belonging to other KOA branches while not working in the southeastern part of Korea. A total of 1,603 participants were enrolled (Fig. 1).

**Table 1:** List of orthopedic resident training hospitals in southeastern Korea

City	Name of Hospital	*Distance (km)
Busan	Dong-A University Hospital	8.9
	Inje University Busan Paik Hospital	4.1
	Inje University Haeundae Paik Hospital	15.0
	Kosin University Gospel Hospital	12.0
	Pusan National University Hospital	9.0
	Pusan National University Yangsan Hospital	25.0
	Bumin Hospital	10.0
	Busan Medical Center	5.0
	Good Samsun Hospital	4.7
Ulsan	Ulsan University Hospital	66.0
Gyeongnam	Gyeongsang National University Hospital	106.0
	Gyeongsang National University Changwon Hospital	36.0
	Samsung Changwon Hospital	52.0

* Distance between hospital and venue of in-person conference

### Transition process to virtual conference

The BUG branch held monthly orthopedic CPD conferences seven times a year before the COVID-19 pandemic [13]. Monthly orthopedic CPD conferences were held in February, March, May, June, September, November, and December, excluding months when national orthopedic conferences by the KOA were held (April and October) and summer vacation periods (July and August). Monthly orthopedic CPD conferences were originally held in the center of Busan Metropolitan City. The 2020 monthly orthopedic CPD conferences could not be held due to the COVID-19 pandemic, but a virtual conference was piloted in September 2020; and held throughout 2021. An equal number of conferences were held in 2019 and 2021. During 2021, the remaining six branches of the KOA did not hold regular orthopedic CPD conferences in the traditional format, nor did they convert to a virtual platform.

### Contents of virtual conference

**Fig. 2:**
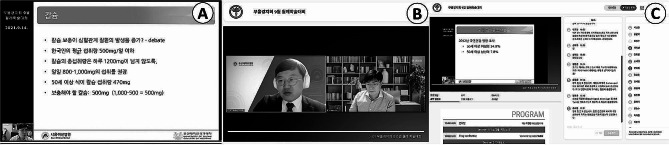
Screenshots from the virtual conference. Speakers presented live on topics or played video file (**A**) with panel discussions (**B**) and audience participation via text chat (**C**)

The traditional, in-person CPD conference is a three-hour event with a single program: an X-ray conference for resident education, a free paper presentation, and an invited lecture. The virtual CPD conference is a four-hour event with a single program featuring experts presenting on a range of topics in four sections: an; each section included live panel discussions and a structured mechanism for audience participation (Fig. 2).

### Format of virtual conference

**Fig. 3:**
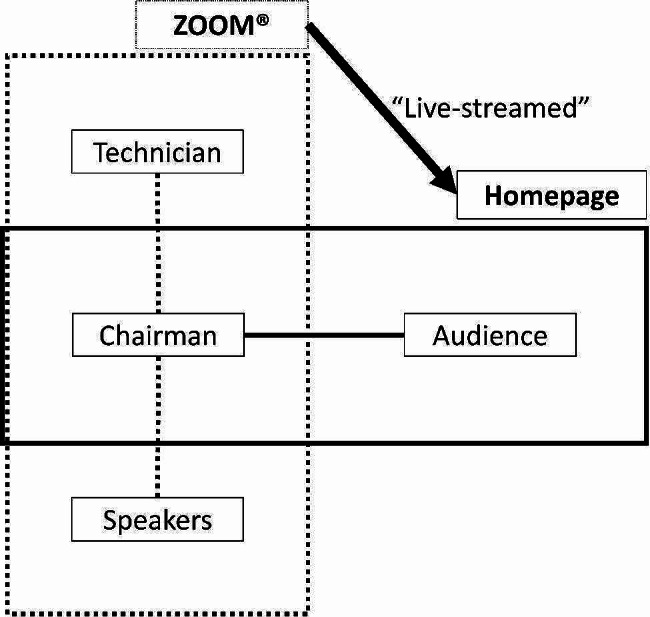
Schematic diagram of platform for new virtual conference

Figure 3 is a schematic diagram of our virtual platform. A webpage for the CPD conference which was linked to the homepage of the regional branch and a cloud-based video communications app (Zoom^®^, USA) were operated simultaneously. In the cloud-based video communication app, the academic director of the regional branch served as the moderator while speakers either presented their lecture live (synchronous content) or played prerecorded lecture files (asynchronous content). Audience members could download the e-book uploaded to the homepage of the BUG branch of the KOA and view the Zoom^®^ lectures and discussions relayed on the website. They could ask questions using a digital messaging platform, and the moderator delivered these questions to the speaker in the Zoom^®^ app, where the speaker could discuss them sequentially.

### Evaluation of participation

We evaluated participation using data that the regional KOA branch collected on its CPD conferences. We counted the participants who attended the VCs using their log-in and log-out records to filter out those who registered but did not attend the conference. We calculated the number of participants for each conference. We counted the number of participants at each VC, classified by the month it was held. Among the information provided by the participants while registering for each conference, we recorded the name of the institution where the participants worked. The locations of the institution where the participants worked were then divided into the central city (Busan Metropolitan City), neighboring city A (Ulsan Metropolitan City), and neighboring city B (Gyeongsangnam-do). The distance between central city and neighboring city A is 41.2 km, and the distance between central city and neighboring city B is 75.7 km. We further classified the institutions by their functionality and size: resident training hospital, general hospital, and private clinic. We compared all variables in the VC year (2021) and IPC year (2019).

We collected data on individual access time on the virtual platform and classified them by 1-hour units to investigate audience participation. We calculated the percentage of participants who accessed 50% or more of the total programs (2 h or more) by month.

A survey was administered to participants who attended the VC held in November or December with the following information. We investigated overall satisfaction with VC and whether applying a virtual platform to these conferences was useful for each item.

### Statistical analysis

Independent *t-*test and chi-square test were carried out using the SPSS version 21.0 (Chicago, IL, USA), and *P* < 0.05 was considered statistically significant.

### Ethics statement

The present study protocol was reviewed and approved by the Institutional Review Board of our institution (approval No. 2022-05-013), which waived the requirement for informed consent due to the retrospective nature of this study.

## Results

### Audience participation

**Fig. 4:**
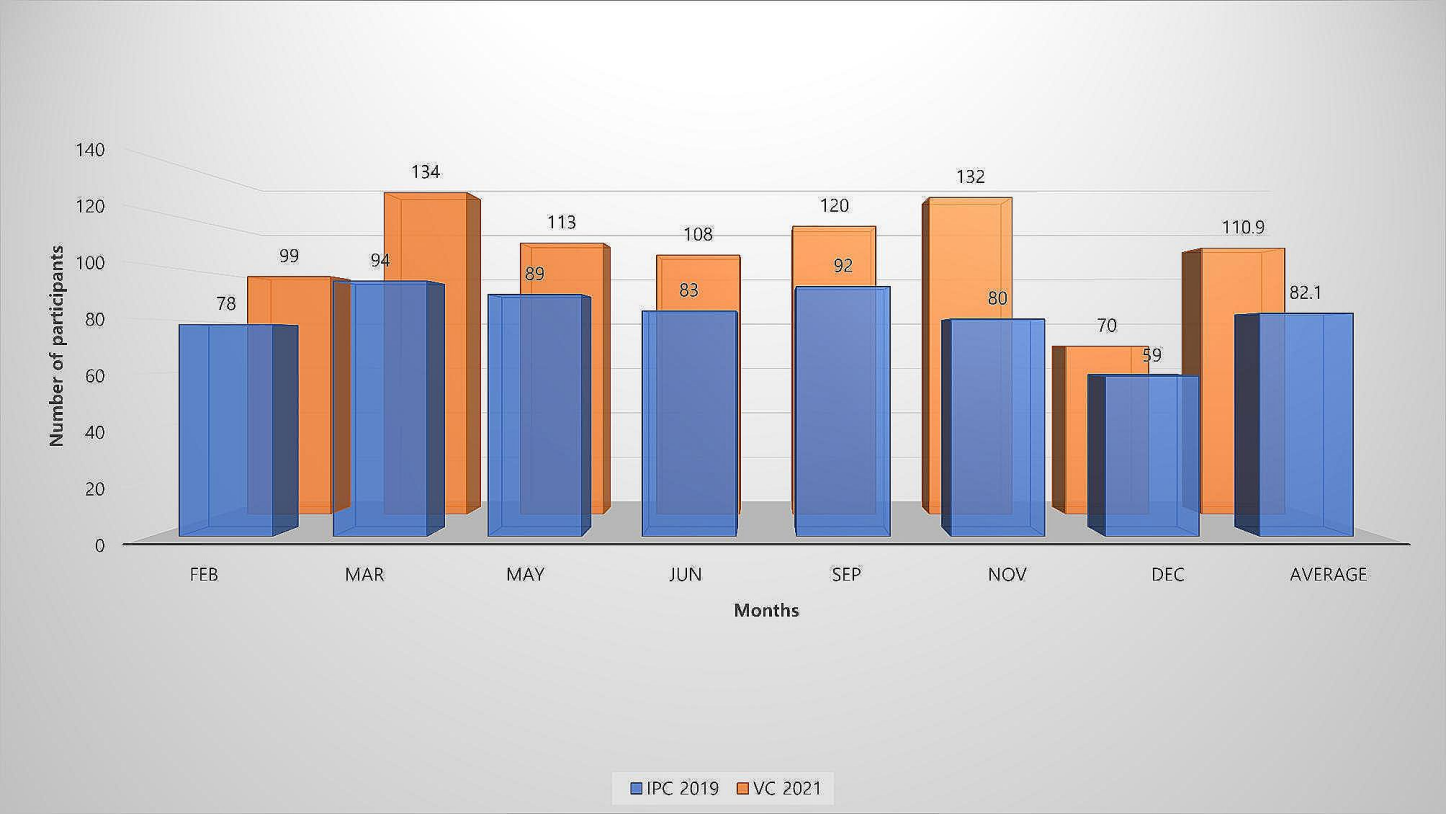
Total number of participants by each conference. IPC 2019, in-person conferences in 2019; VC 2021, virtual conference in 2021

The number of participants who participated in 7 VCs in 2021 was 776, a statistically significant (*P* = 0.01) increase of 201 from the 575 participants in IPCs in 2019. The mean numbers of participants at each VC and IPC were 110.9 (70–134) and 82.1 (59–94), respectively. VCs had more participants than IPCs in each month (Fig. 4).

### Participation according to institution location

**Fig. 5:**
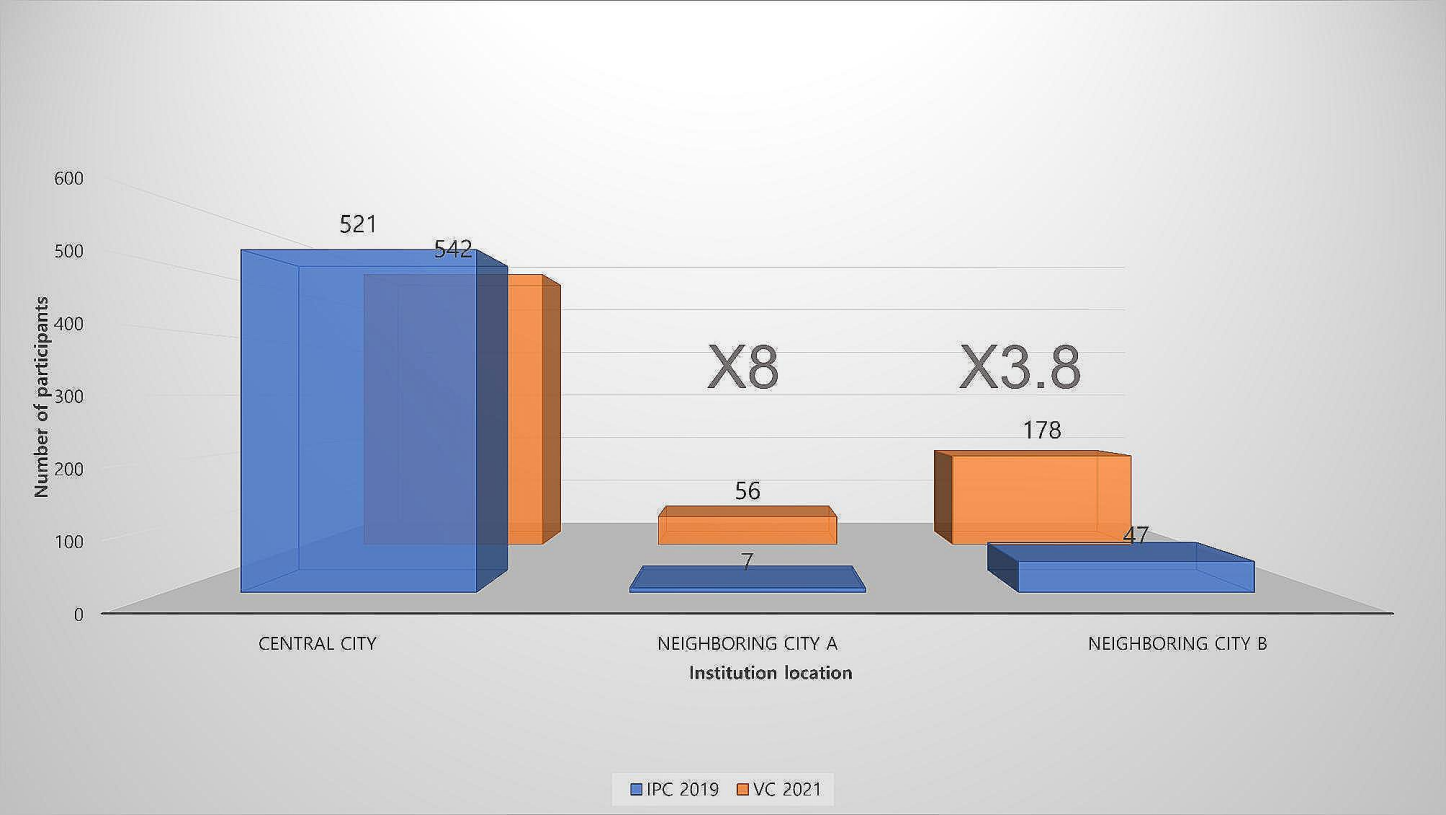
(**A**) Total number of participants by institution location. (**B**) Proportion of participants by institution location in 2019 and 2021

For both the IPCs in 2019 and the VCs in 2021, the region with the largest number of attendees was the central city, with 521 and 542 attendees, respectively. However, the proportion of participants who worked in the central city to the total number of participants decreased from 90.6 to 69.8% (Fig. 5). The number (and proportion) of participants who worked in neighboring city A increased eightfold, from seven (1.2%) during the IPC year to 56 (7.2%) during the VC year (*P* < 0.05). Similarly, the number (and proportion) of participants who worked in neighboring city B increased 3.8-fold, from 47 (8.2%) during the IPC year to 178 (23.0%) during the VC year (*P* < 0.05).

### Participation according to type of institution

**Fig. 6:**
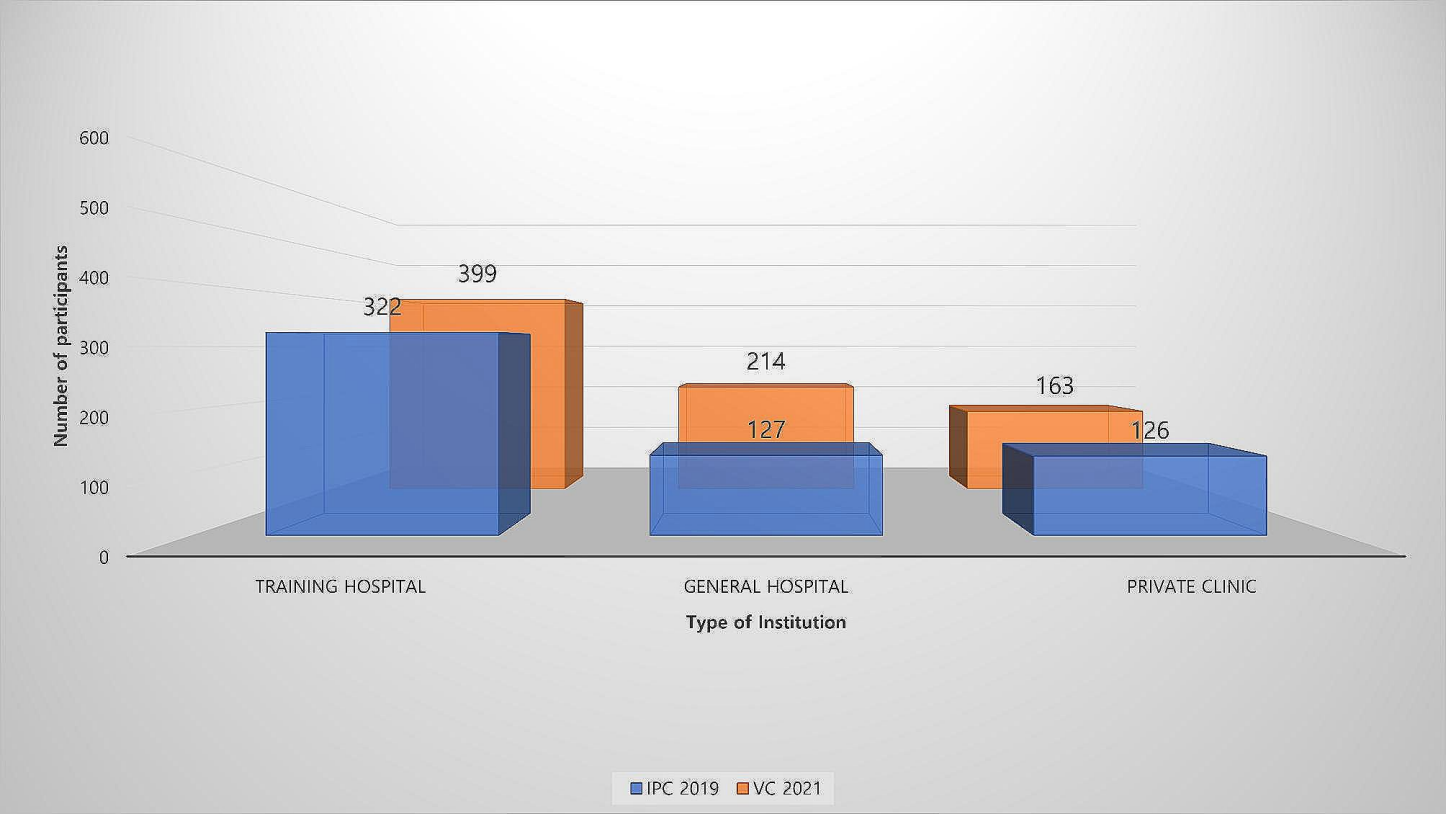
(**A**) Total number of participants by type of institution. (**B**) Proportion of participants by type of institution in 2019 and 2021

For the IPCs in 2019 and the VCs in 2021, the hospitals with the largest number of attendees were resident training hospitals, with 322 and 399 attendees, respectively. However, the proportion of participants from resident training hospitals decreased from 56.0 to 51.4% (Fig. 6). Participants from general hospitals and private clinics increased from 127 to 214 and 126 to 163, respectively. However, only the proportion of participants from general hospitals increased from 22.1 to 27.6%, while the proportion of participants from private clinics decreased from 21.9 to 21.0%.

### Degree of participation in virtual conference

**Fig. 7:**
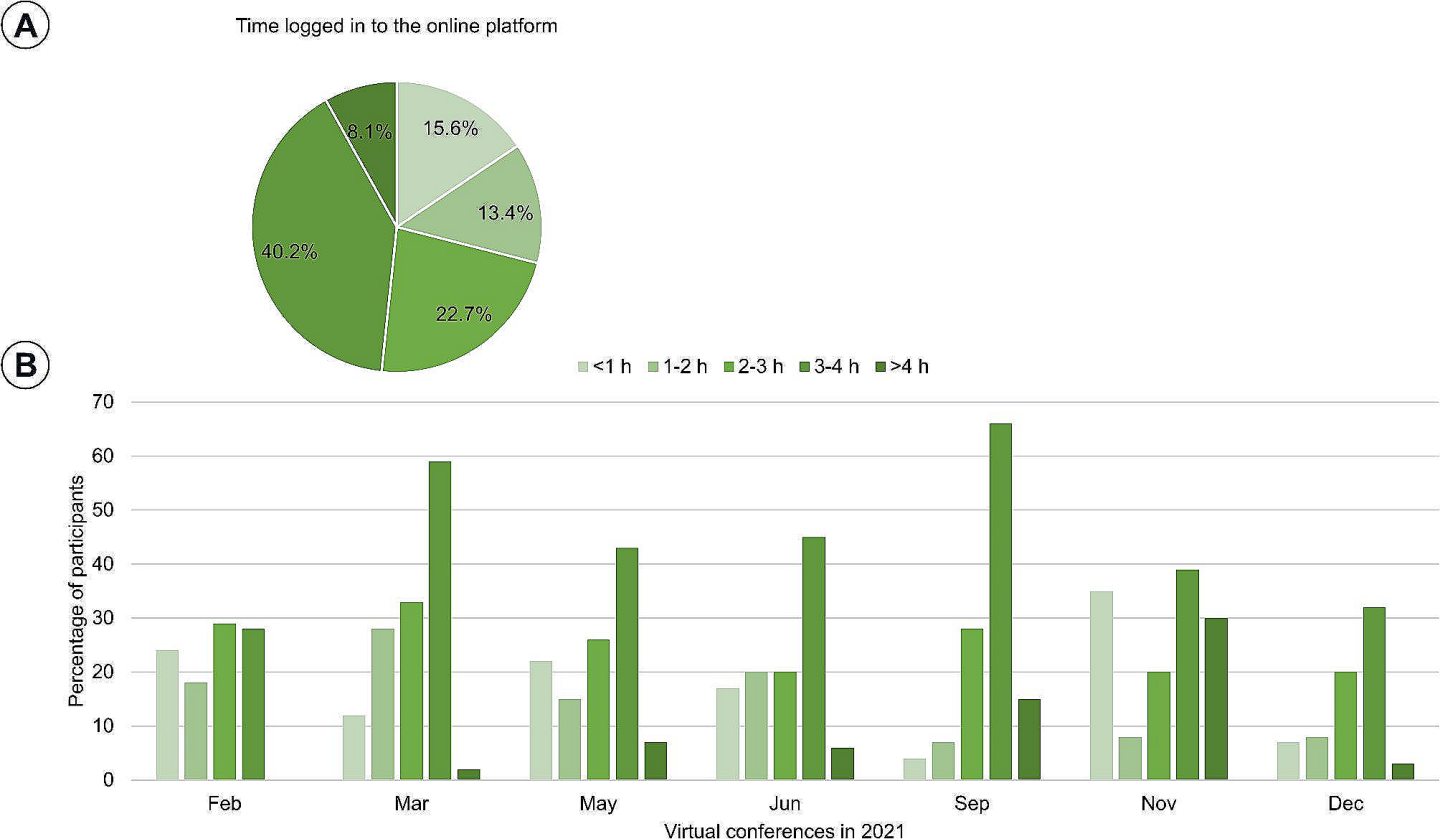
(**A**) Proportion of VC audience member log-in times divided into one-hour intervals (**B**) Distribution of log-in time in each VC

Figure 7A shows the log-in times of VC participants divided into one-hour intervals, showing that 71% of the total audience participated more than half of the educational program. Figure 7B, which shows the log-in time distribution for each VC, shows that out of the four hours of educational time, the proportion of the audience who participated in the educational program for more than three hours and less than four hours was the highest, except for in February.

### Survey on virtual platform application

Table 2 shows the results of a survey on satisfaction with the virtual platform and related items. Conference participants were asked which platform they prefer among platforms for lifelong medical education after pandemic, 36.1% and 22.7% of respondents preferred VC and IPC, respectively, and 41.2% of respondents preferred hybrid conference. As a result of survey on the usefulness of applying the virtual platform for each session, most respondents who answered that application of the virtual platform was useful were in the following order: invited lectures (73.9%), free paper presentations (73.9%), and X-ray conferences (47.1%). In a survey examining the usefulness of the virtual platform for each purpose of the conference, 88.2% of respondents answered that it was useful for interprofessional learning, but less than half of the respondents answered that it was useful for large group discussions (42.8%) and small group workshops (24.4%). In term of personal networking, only 9.2% of the respondents answered that virtual platform was useful.

**Table 2:** Survey on operating a virtual platform

Variables	
Total respondents (n)	119
Survey items	Mean (range)
Overall satisfaction (range, 0–10 points)	7.04 (3–9)
Questions	Yes (n, %)
Q1. What is your preferred platform after the pandemic?	
Virtual conference	43 (36.1%)
Hybrid conference (virtual + in-person)	49 (41.2%)
In-person conference	27 (22.7)%
Q2. Do you think applying the virtual platform for each session is useful?	
X-ray conference for resident education	56 (47.1%)
Free paper presentation	65 (54.6%)
Invited lectures	88 (73.9%)
Q3. Do you think the virtual platform will be useful to each goal of conference?	
Interprofessional learning	105 (88.2%)
Large group discussion	51 (42.9%)
Small group workshops	29 (24.4%)
Personal networking	11 (9.2%)

## Discussion

The present study introduced a virtual platform of monthly orthopedic CPD conferences for regional orthopedic physicians. It showed that the virtual platform increased overall audience participation and increased audience participation from distant cities.

Table 3 shows key points of our virtual CPD conferences. Each virtual conference lasted four hours, from 18:00 to 22:00. Members of our branch consist of orthopedic professionals and trainees working in the central city or neighboring cities A and B. The members of our regional branch can access our virtual CPD conference without registration fees. However, due to requests from orthopedic professionals from other KOA branches, non-members were allowed to participate in the VC after paying the registration fee. During the conference, it was possible to repeat playback on the website, but recorded content was not provided.

**Table 3:** Key points of new virtual platform for orthopedic continuing professional development conferences

Key Points	Sub-variables	Application to our virtual conference
Format	Series of virtual conferences	Seven times a year
	Single live program	(+)
	Panel discussion tools	Zoom^®^
	Audience participation tools	Digital messaging platform
Access	Time	Four hours for each conference
	Members-only	(-)
	Registration fees	Non-members (+)
Industry involvement		E-book
Continuing medical education	Live conference	(+)
	Recorded conference content	Members can download Word or PowerPoint files.

Gottlieb et al. [11] reported that running a VC necessitates considering digital platforms and converting large and small group sessions, abstract presentations, and networking events to a digital medium. Our VC included a section for free paper presentations and provided an important opportunity for project feedback and early dissemination of scholarship. Presenting a free paper at a VC can allow speakers to reach a broader audience while lowering travel costs, minimizing time away from home and work, and avoiding exposure during a pandemic.

The content format of our virtual meeting was freely selectable between live-streamed “synchronous” or prerecorded “asynchronous” depending on the speaker’s preference. This combined approach may be less dynamic but has the benefit of eliminating some of the logistical and technical complexity and risk of delivering a large program with numerous speakers presenting live [14]. We chose this combined approach because the virtual meeting consisted of four sections and at least 6–7 speakers.

CPD conferences provide educational offerings and opportunities to meet other professionals with similar interests. These connections help facilitate intellectual discussions and social support networks. Gottlieb et al. [11] reported that VC coordinators must also provide a mechanism by which participants can connect with their peers. However, our VC platform maintained only one channel for large group lectures and did not operate other channels where participants could gather according to their personal or professional interests.

A survey of the conference participants could aid the evaluation of each session and the conference. This study’s results could be used to further refine topics, identify future speakers, and improve future conference sessions [15, 16]. Previous studies evaluated an overall preference for VCs or IPCs. Residents and faculty who specialized in emergency medicine claimed they missed the social interaction of IPCs and preferred less than 20% of future conferences to be virtual [17]. In a preference study conducted on residents of internal medicine, 42% of residents preferred IPCs, 18% preferred VCs, and 40% felt they were equivalent [18]. In the meantime, there was no overall preference for VCs over traditional IPCs in neurosurgery doctors. Overall, respondents agreed that VCs would partially replace traditional IPCs, but they strongly disagreed that they would completely replace traditional IPCs [19]. Chan et al. [20] compared demographic and survey data on attendance perspectives between the in-person student-led internal medicine conference and the subsequent VC. They reported that, even though the VC was more accessible to attendees, overall learning objectives for the conference and didactic sessions were better met in-person. In a preference survey conducted in this study, 36.1% of respondents to the survey preferred VCs, 41.2% preferred hybrid conferences, and 22.7% preferred IPCs.

As a result of a survey on whether it was useful to apply a virtual platform to X-ray conference session, 47.1% of respondents answered that it was useful. However, in the invited lecture sessions, 73.9% of respondents answered that the application of the virtual platform was useful. Looking at how each session operates; the X-ray conference session was conducted with an educator’s lecture and the educator asking individual questions to the trainee during the presentation. On the other hand, the invited lecture session was conducted in a way that questions could be asked through text chat after the lecture’s presentation was completed. Therefore, it can be interpreted that the differences in the way each session was conducted influenced the evaluation of the usefulness of the virtual platform application.

Participation in lifelong medical education or CPD activities not only provides education-related information, but also provides opportunities for conversations and networking with peers [5]. The virtual platform of this study allowed text-chat between the moderator and the audience to answer questions but did not provide opportunities for conversation between the audience. These limitations of conference operating system appear to be related to the fact that a majority of audience (91.8%) responded that virtual platforms are not useful for personal networking.

In this study, the number of participants who worked in distant neighboring cities increased significantly. The authors speculate that the number of participants in the Ulsan Metropolitan City and Gyeongsangnam-do regions significantly increased in the VC year (2021) because of eliminating the travel distance to the IPC venue. Yates et al. [21] studied whether VCs produced co-benefits for participation and attendee interaction based on five annual international conferences and reported that transitioning online enhanced the geographical reach and attendance of researchers from countries of all income levels. Most of the audience members who participated in the 2021 VC in Ulsan Metropolitan City and Gyeongsangnam-do were physicians at university hospitals, and the authors interpreted that attendance at the CPD conference became easier as the existing restrictions on travel distance disappeared.

Overall, conference attendance at general hospitals and private clinics, which were non-resident education institutions, increased in the VC year (2021). The authors interpreted that the convenience of accessing a virtual platform directly from the hospital space has overcome the factors that previously limited attendance at conferences after work.

The International Microsurgery Club, one of the largest professionals-only online microsurgery education groups worldwide, began hosting regular weekend webinars during the pandemic and reported that webinars fundamentally changed how knowledge was delivered and exchanged [22]. They reported that the number of people requesting to join their society abruptly increased, dramatically increasing group activity. These efforts to transform IPCs into VCs are especially advantageous for organizations whose members are spread out geographically and have trouble attending regional meetings [4, 23, 24].

This study has several limitations. First, we could not analyze audience members’ level of participation and concentration. Although audience participation was possible through digital chat in the system, there were no additional participation methods, such as watching on-demand videos or participating in free discussions in small groups. Finally, we could not analyze the educational outcomes of attendees following the CPD conference. Moore, Green and Gallis provided a paradigm for evaluating medical education on seven levels: participation, satisfaction, declarative and procedural knowledge, competence, performance, patient health, and community health [25]. Follow-up research is needed on not only the audience participation of virtual meetings, but also the knowledge, competence, and performance of CPD in medical education. Nonetheless, this study is the first to analyze the outcomes of virtual CPD conferences conducted over a year, instead of a single conference, during the COVID-19 pandemic. Unlike previous studies on medical education for medical students or residents during the COVID-19 pandemic [4, 17, 18], this study focused on CPD conferences for orthopedic physicians.

## Conclusions

This study presented the results of moving lifelong medical education programs for orthopedic physicians onto a virtual platform during the COVID-19 pandemic by comparatively analyzing in-person and virtual conferences. The virtual platform can be helpful for organizations that must hold regular lifelong medical education programs for members spread across a wide geographic region.

## Data Availability

The datasets used and/or analyzed during the current study are available from the corresponding author on reasonable request.

## Abbreviations

BUG: Busan-Ulsan-Gyeongnam
CPD: Continuing professional development
IPC: In-person conference
KOA: Korean Orthopaedic Association
VC: Virtual conference
